# Determinants of under-five mortality in Ethiopia using the recent 2019 Ethiopian demographic and health survey data: nested shared frailty survival analysis

**DOI:** 10.1186/s13690-022-00896-1

**Published:** 2022-05-13

**Authors:** Belete Achamyelew Ayele, Sofonyas Abebaw Tiruneh, Melkalem Mamuye Azanaw, Habtamu Shimels Hailemeskel, Yonas Akalu, Asnakew Achaw Ayele

**Affiliations:** 1 Amhara Regional Health Bureau, Wogeda Primary Hospital, Wogeda, Amhara Ethiopia; 2 Department of Public Health, College of Health Sciences, Debre Tabor University, Debre Tabor, Ethiopia; 3 Department of Neonatal Nursing, College of Health Sciences, Debre Tabor University, Debre Tabor, Ethiopia; 4 Department of Physiology, College of Medicine and Health Sciences, University of Gondar, Gondar, Ethiopia; 5 Department of Clinical Pharmacy, School of Pharmacy, College of Medicine and Health Science, University of Gondar, Gondar, Ethiopia

**Keywords:** Under-five, Frailty, Ethiopia

## Abstract

**Background:**

Worldwide, there is remarkable progress in child survival in the past three decades. Ethiopia is off-track on sustainable development targets in under-five mortality since 2020. Therefore, this study aimed to investigate time to death and its associated factors among under-five children in Ethiopia.

**Methods:**

Nationally representative demographic and health survey data were used for this study. A total of 5772 under-five children were included. Data were analyzed using R software. Semi-parametric nested shared frailty survival analysis was employed to identify factors affecting under-five mortality. Adjusted hazard ratio (AHR) with 95% Confidence interval (CI) was reported and log-likelihood was used for model comparison. Statistical significance was declared at *P*-value < 0.05.

**Results:**

The weighted incidence of under-five death before celebrating the first fifth year was 5.76% (95% CI: 5.17 – 6.40). Female sex and under-five children living in urban areas were high probability of survival than their counterparts. After controlling cluster and region level frailty, multiple births (AHR = 7.03, 95% CI: 4.40—11.24), breastfed within one hour after birth (AHR = 0.41, 95% CI: 0.28—0.61), preceding birth interval 18–23 months (AHR = 1.62, 95% CI: 1.12 -2.36), and under-five children younger than 18 months (AHR = 2.73, 95% CI: 1.93 -3.86), and teenage pregnancy (AHR = 1.70, 95% CI: 1.01—2.87) were statistically significant factors for time to under-five death.

**Conclusion:**

Even though Ethiopia has a significant decline under-five death, still a significant number of under-five children were dying. Early initiation of breastfeeding, preceding birth interval and teenage pregnancy were the preventable factors of under-five mortality. To curve and achieve the SDG targets regarding under-five mortality in Ethiopia, policymakers and health planners should give prior attention to preventable factors for under-five mortality.

## Background

Worldwide, there is remarkable progress in child survival in the past three decades; which is 1 in 11 children died before the age of five years in 1990 and 1 in 27 children in 2019 [1]. The burden of child death is uneven across regions. Despite this remarkable progress more than half (from 5.2 million deaths) of under-five death was from sub-Saharan Africa (SSA) followed by Central and Southern Asia [1, 2]. Thus, the global under-five mortality rate declined by 59 percent, from 1990 to 38 percent in 2019. Despite this substantial decline, improving child survival remains a matter of urgent concern from preventable child deaths [3].

sub-Saharan Africa is still the region having the highest burden of under-five mortality rate in the world. Of 5.2 million under-five deaths in 2019, more than 80 percent of under-five death reported from SSA and Central and Southern Asia. Half of all under-five deaths in 2019 occurred in five countries, namely Nigeria, India, Pakistan, the Democratic Republic of the Congo, and Ethiopia [1, 4]. The United Nations (UN) Sustainable Development Goal (SDG) 3 target 3.2 calls for reducing under-five mortality to at least 25per 1000 live births by 2030 [5, 6]. In line with this target, most SSA countries are not on track to under-five mortality SDG-3 targets [7]. Only five countries (Kenya, Rwanda, Senegal, Tanzania, and Uganda) are on track to decline the under-5 mortality as per the SDG target [7].

According to the recent commentary evidence, Ethiopia is achieving great child mortality reduction since 2000. This achievement was with financial and technical support from the global partnership. However, the SDG targets for under-five mortality reduction are not achieved by the end of 2019/2020, which might be partnership dynamics due to the COVID-19 pandemic and the pandemic itself might affect the SDG target [8].

Previous studies in Ethiopia tried to investigate the determinants of under-five mortality using the previous demographic and health survey datasets [9–11]. But these studies were not accounting for the survival nature of under-five death and some of the studies ignored the clustering effect of the data. Thus, previous studies had a methodological gap to identify factors affecting under-five death. Demographic and health survey data is nationally representative data collected in two-stage clustering. Modeling of one-level clustering effect or flat models will bias the true effect size of the determinant variables. Therefore, this study was accounting for two-level frailty (nested frailty) survival analysis which controls cluster and region level clustering effects. In advance, previous studies did not control both the cluster and the region level dependency. Thus, this study had an advantage in improving on the methodological gap to get the true effect size factors affecting under-five death. Documenting such, evidence will help the health planners and policymakers to achieve the SDG targets. Therefore, this study aimed to assess time to death and factors affecting under-five death in Ethiopia.

## Methods and materials

### Study setting and data source

This study was conducted on a publicly available dataset on the recent 2019 Ethiopian Demographic and Health Survey (EDHS) data. Demographic and health surveys are nationally representative, community-based cross-sectional household surveys with multi-stage stratified cluster sampling techniques. The details of the sampling and data collection procedure are available on the DHS website (https://dhsprogram.com/).

### Populations and samples

The source population of this study was all live births preceding five years of the survey period in Ethiopia; whereas, the study populations were all live births preceding five years of the survey period in the selected Enumeration Areas (EAs). The required sample size was extracted from the children's record (KR) file from the standard DHS dataset. Finally, a total of 5772 under-five children were included in this study.

### Study variables

#### Outcome variable

Time to under-five death in months.

#### Independent variables

The independent variables were socioeconomic and demographic factors (mothers and husband education, wealth index of the household, place of residence, latrine, and water source availability), maternal reproductive and obstetric characteristics (preceding birth interval, mothers age at first birth, place of delivery, antenatal care visit, parity, and birth order), and infant characteristics (sex of the child, weight of the child at birth, plurality of the child).

### Operational definitions

**Time to under-five death:** The time of under-five death before 60 months.

**Event:** The death of under-five children before celebrating their five years of birthday.

**Censored**: Under-five children did not experience the event of interest before 60 months.

### Data management and analysis

The data were cleaned and coded using STATA version 16/MP software. The extracted data were imported to R version 4.0.5 software for analysis. The descriptive statistics were reported in percentages and tables. Kaplan Meier non-parametric survival analysis was employed to estimate conditional probabilities each month when an event occurs.

### Modeling of semi-parametric nested shared frailty survival analysis

To identify the independent determinants of under-five death, Cox proportional hazard nested shared frailty survival model was employed. Frailty models are the hazard models having a multiplicative frailty factor. This factor specifies how frail subjects in a specific cluster or region are [12]. Therefore, this study assumes and controls the frailty factor at both the cluster and region levels. Under-five children are nested in the clusters and clusters nested in the regions of the country. This nested frailty model for two-level (clusters nested in regions) hierarchically clustered was fitted using *frailty pack R*-package. The model structure of this hierarchical survival model is.

$${h}_{ijk}\left(t\right)={h}_{0}\left(t\right){u}_{i}{z}_{ij} {\mathrm{exp}(}_{ijk}^{t}\beta )$$

Where h_ijk_(t) the hazard of under-five death at time t of subject k, k = 1,..., nij clustered in the j^th^ cluster, j = 1,..., ni, of region i, i = 1,..., n; h_0(t)_ is the baseline hazard function, U_i_ the frailty term for the region level and Z_ij_ the frailty term for the cluster level. The i^th^ term in the region level i, i = 1,..., s; u_i_ is the frailty term for region i and z_ij_ is the frailty term for cluster j nested in the region i. We assume the frailty terms independent and gamma distribution for the random effect frailty term and maximum marginal likelihood using penalized log-likelihood for the baseline hazard. The proportional hazard semi-parametric cox regression was used to estimate the fixed effect estimation.

Three models namely cluster, region, and nested (cluster nested to region) level frailty model was fitted. The best fit model was selected using the marginal penalized log-likelihood ratio test was used. The lowest penalized log-likelihood was the best fit model.

### Ethical consideration

This study was conducted after securing a waiver of written informed consent from the International Review Board of Demographic and Health Surveys (DHS) program data archivists. The dataset is publicly available in requesting a concept note for a proposed project.

## Results

### Sociodemographic characteristics of the respondents

Overall, a total of 5772 study participants were included in this study. The majority 4,653 (80%) of the study respondents (mothers) age were in the age category of 20–34 years. Overall, 749 (13.53%) of the mothers had teenage pregnancies. Almost of the mothers were married and three fourth of the mothers were living in rural areas. More than half (53.82%) of the mothers had no formal education and 45.84% of the household were poor household wealth status. Surprisingly, only 11.44% of the household had improved water sources and 4.56% of the household had flush latrines (Table 1).

**Table 1:** Maternal and socio-economic background characteristics of the study respondents' recent EMDHS 2019, 2021

Variables	Categories	Frequency (n)	
		**Unweighted, n (%)**	**Weighted, n (%)**
Maternal age	15–19	297 (5.15)	263 (4.75)
	20–34	4,653 (80.61)	4455 (80.41)
	35–49	822 (14.24)	822 (14.83)
Mother’s age at birth	≥ 20 years	4881 (84.56)	4791 (86.47)
	≤ 19 years	891 (15.44)	749 (13.53)
Marital status	Not union	31 (0.54)	22 (0.41)
	Union	5741 (99.46)	5518 (99.59)
Mother educational status	No education	3169 (54.90)	2982 (53.82)
	Primary	1823 (31.58)	1950 (35.19)
	Secondary and above	780 (13.51)	609 (10.59)
Residence	Urban	1328 (23.01)	1367 (24.67)
	Rural	4444 (76.99)	4173 (75.33)
Head of household	Male	4618 (80.01)	4792 (86.49)
	Female	1154 (19.99)	748 (13.51)
Wealth status	Poor	2976 (51.56)	2539 (45.84)
	Middle	803 (13.91)	1037 (18.72)
	Rich	1993 (34.53)	1963 (35.44)
Latrine facility	Flush latrine	319 (5.53)	256 (4.56)
	VIP / traditional latrine	2952 (51.14)	3370 (60.82)
	No latrine	250 (43.33)	1915 (34.56)
Water source	Improved	801 (13.88)	634 (11.44)
	Unimproved	4971 (86.12)	6906 (88.56)
**Total**		**5772**	**5540**

### Characteristics of the study participants

Among the total of under-five children, more than half (51.55%) were males and 145 (2.65%) of them were multiple births. Most of 3311 (77%) of under-five children were older than two years. Overall, 2745 (49.55%) of under-five children did not immediately breastfeed after birth. Almost forty-eight percent of under-five children were delivered at the health facility and 5% of them were delivered by cesarean section (Table 2).

**Table 2:** Background characteristics of under-five children among recent 2019, EMDHS data, and 2021

Variables	Categories	Frequency (n)	
		**Unweighted, n (%)**	**Weighted, n (%)**
Infant sex	Male	2985 (51.72)	2855 (51.55)
	Female	2787 (48.28)	2685 (44.47)
Plurality	Single	5604 (97.09)	5393 (97.35)
	Multiple	168 (2.91)	145 (2.65)
Preceding birth interval	≥ 24 months	3374 (75.26)	3311 (76.75)
	18–23 months	609 (13.58)	605 (14.02)
	< 18 months	500 (11.15)	398 (9.23)
Birth order	≤ three	3171 (54.94)	2981 (53.81)
	≥ four	2601 (45.06)	2559 (46.19)
Breastfeeding at birth	Not-immediately	3009 (52.13)	2745 (49.55)
	Immediately	2763 (47.87)	2795 (50.45)
ANC visits	No ANC	2860 (49.55)	2636 (47.57)
	At least one ANC	2912 (50.45)	2904 (52.43)
Place of delivery	Health facilities	2789 (48.76)	2622 (47.70)
	Home delivery	2931 (51.24)	2875 (52.30)
Mode of delivery	SVD	5424 (93.97)	5241 (94.60)
	Cesarean section	348 (6.03)	299 (5.40)
**Total**		**5772**	**5540**

### Kaplan Meier survival analysis

**Fig. 1:**
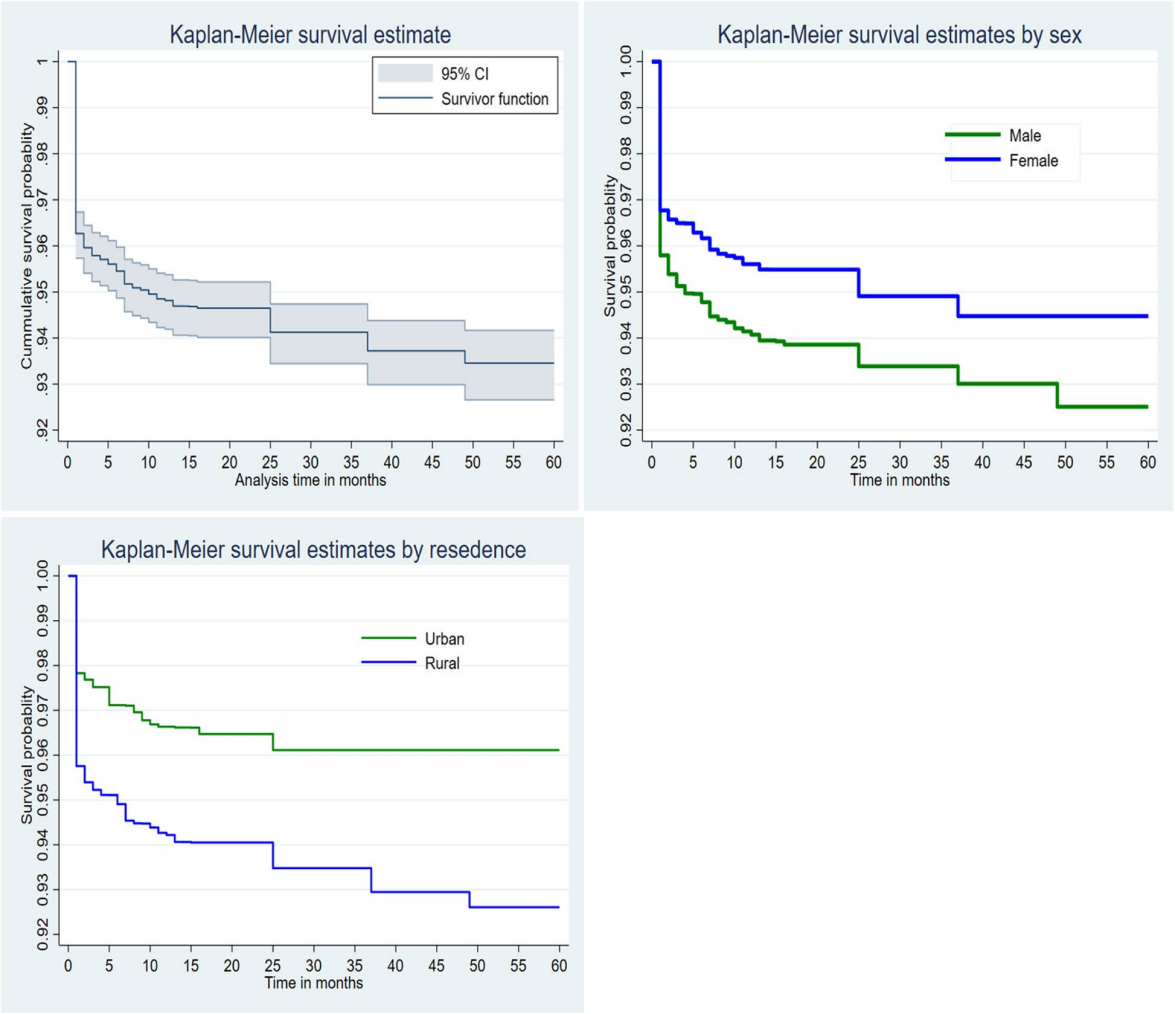
Overall Kaplan Meier survival estimate under-five death, survival estimates by sex, and survival estimates by residence among under-five children in Ethiopia

The cumulative survival probability of under-five children sharply decreased in the first month of life. After the first month of life, the probability of survival of infants decreased proportionally. Female under-five children had higher survival status than male under-five children. Infants living in rural areas had a high probability of death than their counterparts (Fig. 1).

### Factors affecting under-five death

Nested shared frailty Cox regression survival analysis was fatted to identify factors affecting under-five mortality. These cluster and region-level dependency accounts for the dependency (frailty) of under-five death in Ethiopia. The frailty with the cluster and region level was statistically significant. The cluster-level frailty theta was 0.91 (*P*-value, < 0.001) and region level frailty theta was 0.007 (*P*-value, 0.009).

Comparing cluster, region, and both cluster and region level frailty the nested shared frailty model (both region and cluster) was the best fit model which has the lowest marginal log-likelihood (-1159.67). From the nested frailty model multiple births, breastfeeding within one hour after birth, preceding birth interval and teenage pregnancy were statistically significant predictors for under-five death.

Controlling cluster and region level dependency, under-five children who had multiple births were seven times more likely at risk of death as compared to singleton birth (AHR = 7.03, 95% CI: 4.40—11.24). The hazard of death among under-five children who had breastfed within one hour after birth was decreased by 59% as compared to their counterparts (AHR = 0.41, 95% CI: 0.28—0.61). The hazard of death among under-five children born within 18–23 months preceding birth interval increased by 62% as compared to under-five children born older than two years preceding birth interval (AHR = 1.62, 95% CI: 1.12 -2.36). Besides, under-five children born younger than 18 months preceding birth interval were 2.73 times more likely to die than under-five children born older than two years preceding birth interval (AHR = 2.73, 95% CI: 1.93 -3.86).

Furthermore, the hazard of death among under-five children who had been born to teenage mothers was 70% more likely to die than under-five children born from their counterparts (AHR = 1.70, 95% CI: 1.01—2.87) (Table 3).

**Table 3:** Nested shared frailty survival analysis factors affecting under-five death using the recent 2019 MEDHS data. (*n* = 5772)

Valuables		Cluster level frailty (Model 1)	Region level frailty (Model I1)	Nested frailty (Model II1)
		**AHR (95% CI)**	**AHR (95% CI)**	**AHR (95% CI)**
Child sex	Male	1	1	1
	Female	0.86 (0.66—1.14)	0.85 (0.65—1.11)	0.86 (0.65 -1.13)
Plurality	Single	1	1	1
	Multiple	6.72 (4.23—0.69)	5.54 (3.70—8.30)	7.03 (4.40 -11.24) ***
Immediate breastfed status	> 1 h	1	1	1
	≤ 1 h	0.41 (0.28—0.60)	0.44 (0.30—0.64)	0.41 (0.28—0.61) ***
Birth interval	≥ 24 months	1	1	1
	18–23 months	1.63 (1.12 -2.37)	1.65 (1.15—2.38)	1.62 (1.12 -2.36) *
	< 18 months	2.74 (1.94—3.87)	2.85 (2.05—3.95)	2.73 (1.93 -3.86) **
Birth order	≤ three	1	1	1
	≥ four	1.31 (0.94—1.83)	1.27 (0.92—1.75)	1.32 (0.95 -1.84)
Teenage pregnancy	No	1	1	1
	Yes	1.68 (1.00—2.84)	1.52 (0.92—2.51)	1.70 (1.01—2.87) *
Place of delivery	Health facility	1	1	1
	Home	0.93 (0.66 -1.33)	1.04 (0.75—1.44)	0.94 (0.66 -1.34)
ANC visit	No	1	1	1
	At least one ANC	0.75 (0.51 -1.10)	0.74 (0.51—1.08)	0.75 (0.51—1.10)
Maternal education	No education	1	1	1
	Primary	1.21 (0.86 -1.69)	1.22 (0.89—1.67)	1.21 (0.86—1.68)
	Secondary & above	0.62 (0.28 -1.33)	0.61 (0.29—1.26)	0.62 (0.28—1.33)
Residence	Urban	1	1	1
	Rural	0.78 (0.45—1.36)	0.73 (0.47—1.14)	0.77 (0.44—1.35)
Household wealth index	Poor	1	1	1
	Middle	1.25 (0.79—1.97)	1.19 (0.78—1.82)	1.25 (0.79—1.96)
	Rich	1.30 (0.82—2.04)	1.12 (0.74—1.68)	1.29 (0.82—2.04)
Water source	Improved	1	1	1
	Unimproved	1.46 (0.72 -2.96)	1.51 (0.80—2.85)	1.47 (0.73—2.99)
**Penalized log-likelihood**		**-1196.98**	**-1216.26**	**-1159.67**
**Region level frailty θ (p-value)**			**0.036 (0.2)**	**0.007 (0.009)**
**Cluster level frailty θ (p-value)**		**0.88 (< 0.001)**		**0.91 (< 0.001)**

NB: *** = significant at 0.001 level, ** = significant at 0.01 level, * = significant at 0.05 level, *θ* theta, *AH* Adjusted Hazard Ratio, *CI* Confidence Interval

## Discussion

Under-five mortality is an important indicator for child survival as well as the all-over well-being of the population at large [13]. This study revealed that the weighted incidence of under-five mortality was 5.76% in Ethiopia, which is higher than from average world index (3.4%) and nearly similar to the previous 2016 Ethiopian demographic and health survey report but lower than a study done in Chad (13%) [14–16]. The possible source of variation might be due to country’s socioeconomic status difference, universal health coverage, time of the study, and study setting difference.

This study tried to identify factors affecting under-five mortality. Multiple under-five births were seven times more likely to die than singleton births. This finding is supported by previous studies conducted in Kenya [17] and the United States [18]. The possible justification might be biological factors [19], multiple births might have susceptible to obstetrics, perinatal and other risk factors, that determine the under-five mortality [17, 20]. Besides, multiple births increase individual family size which leads to prenatal attention per child diminishing [21].

Controlling other variables constant, the hazard of death among under-five children who had breastfed within one hour after birth was decreased by 59% as compared to their counterparts. This means early initiation of breastfeeding will bring a good survival among children. This finding is supported by previous studies [17, 22, 23]. This might be because early initiation of breastfeeding within the first hour can help prevent under-five death from different infections and reduce morbidity [24]; since**,** early initiation of exclusive breastfed served as the starting point for continuum care for the mother by promoting the bonding, providing colostrum as baby’s first immunization and newborn that can have a long-lasting effect on health and brain development for beyond [25]. As well due to traditional and cultural malpractice, women may avoid colostrum breastfeeding, which inhabits the source of immunoglobulin g for their children [22].

Based on this study, the hazards of death among under-five children born within 18–23 months preceding birth interval increased by 62 percent as compared to under-five children born more than two years. Evidence from a previous study indicates that a short birth interval had a risk of under-five survival [26]. This study is also, supported by a study done on Afghanistan [27]. Having many children who have a short preceding birth interval, increase the vulnerability of children; it might be explained that, closely spaced family prone to overcrowding, poor child care and food insecurity, and hygienic problem leads to under-five children morbidity and mortality may happen [19].

Moreover, after adjusting for known confounding factors and cluster and region level frailties, under-five children who were born from teenage mothers were 70% times more likely to die than children born from their counterparts. This finding is in line with previous studies conducted in Kenya [17], Uganda [28], Ethiopia [29] and Afghanistan [27]. Maternal age, directly and indirectly, affects under-five mortality. The majority of teenage pregnancies happened unwanted and unsupported which caused maternal mental, psychological and social problems, interns of lack of child care, support, and feeding that could bring under-five mortality [30]. In conclusion, we identified independent factors, namely; multiple births, early initiation of breastfeeding, preceding birth interval, and teenage pregnancy for under-five mortality in Ethiopia.

This study follows some limitations and strengths. Since the study was conducted based on a nationally representative large dataset that could enhance the generalizability of the estimates. As well this study was controlling cluster level and region level dependencies using nested shared frailty survival analysis that gives the unbiased true effect size for the determinant factors. However, the data were collected cross-sectionally, which would be prone to recall and social desirability bias. The drawback of the secondary nature of data was inevitable.

## Conclusion

Even though Ethiopia has a significant decline in under-five death, still a significant number of under-five children were dying. Early initiation of breastfeeding, preceding birth interval and teenage pregnancy were the preventable factors of under-five mortality. As well, multiple pregnancies were a significant predictor of under-five mortality. To curve and achieve the SDG targets regarding under-five mortality in Ethiopia, policymakers and health planners should give prior attention to preventable factors for under-five mortality.

## Data Availability

The data were publicly available datasets in the DHS program.

## Abbreviations

AHR: Adjusted Hazard Ratio
CI: Confidence Interval
EMDHS: Ethiopian Mini Demographic and Health Survey
SSA: Sub-Sharan Africa
SDG: Sustainable Development Goals
